# Biogenic Silver Nanoparticles: Synthesis and Application as Antibacterial and Antifungal Agents

**DOI:** 10.3390/mi12121480

**Published:** 2021-11-29

**Authors:** Artem Rozhin, Svetlana Batasheva, Marina Kruychkova, Yuliya Cherednichenko, Elvira Rozhina, Rawil Fakhrullin

**Affiliations:** Institute of Fundamental Medicine and Biology, Kazan Federal University, Kreml uramı 18, 420008 Kazan, Republic of Tatarstan, Russia; rozhinartemkzn@gmail.com (A.R.); svbatasheva@gmail.com (S.B.); maricshka80@gmail.com (M.K.); serova.yuliya87@gmail.com (Y.C.)

**Keywords:** nanoparticles, biogenic silver nanoparticles, antimicrobial properties, self-assembly of nanoparticles, bio-fabrication

## Abstract

The importance and need for eco-oriented technologies has increased worldwide, which leads to an enhanced development of methods for the synthesis of nanoparticles using biological agents. This review de-scribes the current approaches to the preparation of biogenic silver nanoparticles, using plant extracts and filtrates of fungi and microorganisms. The peculiarities of the synthesis of particles depending on the source of biocomponents are considered as well as physico-morphological, antibacterial and antifungal properties of the resulting nanoparticles which are compared with such properties of silver nanoparticles obtained by chemical synthesis. Special attention is paid to the process of self-assembly of biogenic silver nanoparticles.

## 1. Introduction

**Figure 1:**
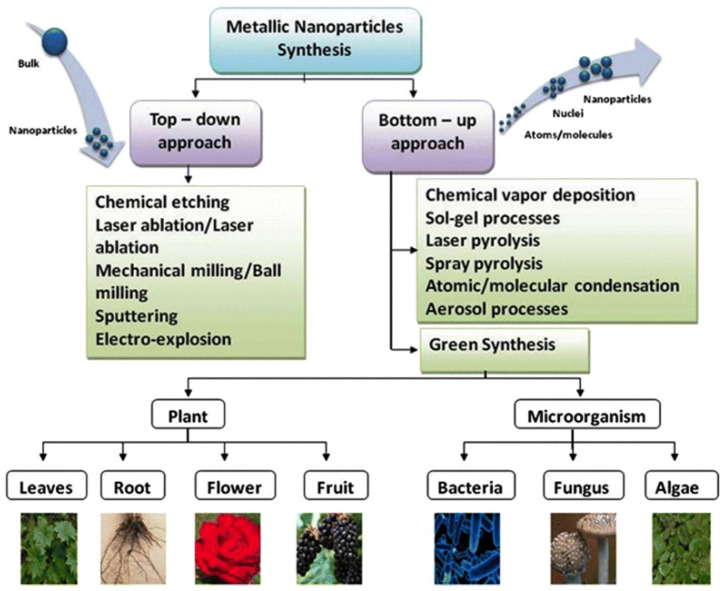
Example of the use of various approaches and biological objects to obtain metal nanoparticles. Demonstration of various synthesis methods for the metal nanoparticles [5] The materials are licensed under http://creativecommons.org/licenses/by/4.0/ (accessed on 28 November 2021).

Today, developments in the nanotechnology field draw great attention due to the use in many fields of science of various nanomaterials, including nanoparticles. Nanomaterials have deserved this interest because of their unique size, physico-chemical properties, and activity in biological systems. The unique properties of metal nanoparticles find application in biological labeling, drug delivery, diagnostics, imaging, probing, gene insertion, artificial implant production, and tissue engineering [1]. Recent advances in nanotechnology show that nanomaterials, in particular silver-based ones, can play a crucial role in biological, pharmaceutical and biomedical fields. The size of nanoparticles significantly affects their properties (electrical, magnetic, toxic, etc.), which determines the importance of the synthesis process for obtaining nanoparticles of a given shape and size (Figure 1). Nanoparticles of certain morphology can be synthesized by self-assembly of Ag ions on supramolecular supports, and applied in such areas as biochemistry, catalysis, biosensors, and microelectronics [2].

Among the variety of metal nanoparticles, silver nanoparticles (AgNPs) have found wide application in various fields due to their superior physical, chemical and biological characteristics. Research in this area is aimed at finding new simplified methods for obtaining nanomaterials and their use in various fields of human activity. Biomimetic technologies are environmentally friendly and economically viable for the synthesis of materials [3]. Three main components are involved in the production of nanoparticles using biological methods: a solvent medium, an environmentally friendly reducing agent, and a non-toxic stabilizing agent [4]. The synthesis of nanoparticles using parts of plants, algae, fungi, and microorganisms has an advantage over chemical synthesis because the latter can produce toxic compounds adsorbed on a nanostructured surface, hindering the use of chemically synthesized particles for medical purposes [5]. It was also found that biogenic objects secrete a large amount of proteins that contribute to the reduction of metal ions and allow the control over the morphology of the resulting particles. At the same time, an important issue is the disposal of nanomaterials after their intended use, since, for example, the release of silver nanoparticles (AgNPs) into the aquatic environment can pose a potential risk to humans and other organisms when their levels exceed safe permissible levels [6]. Currently, there are a number of examples of the successful application of reusable functional products containing silver nanoparticles. AgNPs immobilized on cotton cloth were used as a catalyst for the reduction of nitroaromatics [7]. It has been shown that the catalyst can be recycled up to 6 times without significantly reducing its catalytic efficiency. A new nanocatalyst based on fibrous nanosilica with a high surface area containing silver nanoparticles dispersed on microsphere fibers has also been successfully developed [8]. The synthesized nanocatalyst was easily recovered and reused for at least 10 cycles. Veisi also notes that the AgNPS-based nanocatalyst can be isolated from reaction solutions and processed without losing its high efficiency [9].

Thus, biogenic synthesis of nanoparticles is economically efficient and safe. However, there is no orderly compiled data on the self-assembly of biogenic silver nanoparticles. It is necessary to collect new data on biogenic AgNPs, in particular, to compare the properties of silver nanoparticles obtained by different methods. The review also considers recent works on the antibacterial and antifungal properties of biogenic silver nanoparticles.

## 2. Synthesis and Self-Assembly of Biogenic Silver Nanoparticles

The properties of AgNPs depend on their size, shape, and morphology [10]. Different ways can be used for the synthesis of silver nanoparticles, namely chemical, physical and biological synthesis. Chemical method for AgNPs synthesis in solution requires reducing agents and a stabilizing agent. Physical synthesis of AgNPs includes the evaporation-condensation method and the laser ablation method [11]. Physicochemical synthesis, as a rule, is more laborious and hazardous than biological synthesis of AgNPs, which has emerged as an alternative approach and has a number of advantages [12]. Biogenic nanoparticles are safer and more environmentally friendly, since the synthesis process occurs at normal temperature and ambient pressure [13]. Additionally, the biomass used for nanoparticle synthesis is easily handled and utilized [14,15]. Thus, biological methods can be advantageous over physical and chemical methods of synthesis, such as thermal evaporation, ultrathin films method, lithography technique, diffusion-flame synthesis [16], sol—gel process, electrodeposition, chemical vapor deposition [17], chemical solution deposition, hydrolysis [10], catalytic method and coprecipitation method. Biogenic silver nanoparticles obtained using various parts of plants, fungi, microorganisms and algae have excellent self-assembly properties and exhibit much the same properties as AgNPs synthesized by chemical and physical methods.

The physicochemical properties of silver nanoparticles are largely determined by the synthesis conditions: temperature, reaction time, pH, molar ratio of reagents, order of addition of salts and reducing agents, speed and type of stirring, etc. [18]. For example, the AgNPs with a size of 18 ± 4 nm were synthesized at a temperature of 120 °C and a heating rate of 7.5 °C min^−1^ by the precursor heating method, where the main factor affecting particle size was heating rate. The using of the precursor injection method and temperature of 100 °C allowed the authors to obtain the silver nanoparticles with a size of 17 ± 2 nm [19]. At the same time the maximum synthesis of biogenic silver nanoparticles prepared with stem extract of *Cissus quadrangularis* was achieved within 1 h, at pH 8 and temperature of 70 °C [20]. In addition, to obtain small monodisperse nanomaterials, it is important that the nucleation process takes place in a short period of time [19]. Nanoparticles synthesized using adsorption interaction with resorcinol in an alkaline medium demonstrated significant differences in physical and chemical properties depending on the preparation conditions that affected the nanoparticle morphology, size and aggregation [21]. It has been shown that the production of nanoparticles of a certain shape and size can be achieved by changing the methods of synthesis, reducing agents and stabilizers [22]. The Kundu et al. was showed that Ag nanochains formation was raised by low TX-100 concentrations and high pH [23]. The reduction of silver particles occurs, as a rule, in the presence of stabilizers, which play an essential role in determining the particle size and shape, as well as ensuring their stability [24]. Recently, various types of surfactants have begun to be used as stabilizing agents in the synthesis of AgNPs to increase their stability, such as sugars [25], trisodium citrate [26], lignosulfates [27], ascorbic acid [28], plant extracts [29] etc. Cationic surfactants have a powerful stabilizing effect on AgNP by binding to the surface of silver nanoparticles through chemisorption. For example, it has been shown that the stabilization of silver nanoparticles with DTAB (dodecyltrimethylammonium bromide), TTAB (tetradecyltrimethylammonium bromide) and CTAB (hexadecyltrimethylammonium bromide) is very effective, and the stabilizing effect is increased by increasing the length of hydrophobic substituents on the polar ammonium head, which results in the formation of small sized nanoparticles with high positive zeta potential values [30]. Moreover, some nonionic surfactants or leaf extract can act not only as stabilizers, but also as reducing agents [29,31]. For example, the use of the surfactant Tween 80 is described for the synthesis and self-assembly of stable water-soluble silver nanoparticles, which acted simultaneously as a reducing agent and a stabilizer, which is certainly more environmentally friendly. In addition, this method provides a high concentration of silver colloids and can be applied in industrial production [32]. In the study of Behravan et al. the influence of AgNO_3_ concentration, extract concentration of *Berberis vulgaris* and reaction incubation time were identified for the biosynthesis of AgNPs [18].

The question of whether there are significant differences in shape, size, and polydispersity between silver nanoparticles obtained by chemical and biological methods seems to be quite interesting. A number of authors claim that the size of biogenic AgNPs is within 20–25 nm [33,34]. In the work of Spagnoletti it is indicated that the average size of biogenic silver nanoparticles (40 nm) was larger than that of chemically synthesized particles (20–30 nm) [35]. He noted that, with both types of nanoparticles being spherical, chemical AgNPs were more uniform in shape and highly dispersed, while biologically produced AgNPs formed agglomerates. In another work, sizes within 35 ± 10 nm, a spherical shape, and uniformity with a polydispersity index of 0.337 for biologically synthesized silver nanoparticles were shown [36]. Such fragmentary data from different authors do not give a general picture and sometimes contradict each other, which may mean that the entire set of synthesis conditions is important for these characteristics, and that multiple factors can influence the results. These assumptions are confirmed by some studies comparing AgNPs synthesized under different conditions; for example, it was shown that at pH 6, the average size of biogenic particles was 42.4 nm, while at pH 11, the particles had an average size of 21.4 nm and were monodisperse [37]. The pH of the solution affects not only the size, but also the shape and polydispersity of the obtained AgNPs [38]. Nanoparticles formed at pH 3 have different morphologies, such as rods, triangles, spheres, and other irregular shapes, while nanoparticles obtained at pH 5 to 7 have a predominantly spherical shape with a relatively uniform size distribution. At pH 9, a mixture of spherical and elongated nanoparticles is formed [38]. In addition to the acidity of the medium, other factors such as temperature and reaction time can be manipulated in biological synthesis. The effect of three different pH values 5.0, 7.0 and 9.0 at different temperatures and reaction times was observed when manipulating the silver nanoarchitecture [39]. Time has played an important role in the synthesis of spherical, penta/hexagonal and rectangular nanoparticles, along with pH and temperature. The particle size also increased from homogeneous nanoparticles with a size of 2–5 nm at 24 h (pH 7, 30 °C) to particles with a size of up to 80 nm after 72 h of incubation. The same results were obtained at pH 5 under all different reaction conditions, however, at pH 9, with an increase in the time interval from 24 to 72 h, the shape of silver nanoparticles changed from spherical to mixed, consisting of spherical, triangular and rectangular particles at 40 °C [39]. Thus, it turned out that there is not a single factor that controls the conversion of biogenic silver nanoparticles into different shapes and sizes, it is a unique balance of various interacting physical parameters. In chemical synthesis, the architecture of silver nanoparticles can be controlled using reducing agents such as poly (N-vinylpyrrolidone), polyacrylic acid, and capping with several organic solvents [39]. There have also been successful attempts to obtain various shapes and sizes of silver nanoparticles by chemical synthesis by varying the reaction time and stirring time. Thus, there is certain variability in the shape, size, and polydispersity of silver nanoparticles synthesized by both chemical and biological methods.

The presence of a capping agent in biogenic AgNPs protects particles from aggregation [35], which to a certain extent improves their stability. Biological molecules perform a double function—the reduction during the synthesis and stabilization of silver nanoparticles in water [33]. It was also noted that nanoparticles formed with the help of microorganisms were very stable and persisted without the formation of aggregates for 90 days [36]. Green synthesis of stable silver nanoparticles at room temperature has been demonstrated using the leaves of *C. angustifolia* [37]. A solution of biogenic silver nanoparticles obtained using plant extracts was stable for two months [33]. There is also a mention of the long-term stability of green AgNPs obtained using an extract of the alga *Parachlorella kessleri* (syn. *Chlorella kessleri*) [6]. AgNPS did not have direct contact with each other even inside small aggregates. In addition, the effect of the synthesis conditions on the stability of silver nanoparticles was shown [40]. Particles obtained under alkaline conditions were relatively more stable than those under acidic conditions; long-term experiments with extended synthesis times also contributed to stability, while rapid processes for preparing AgNPs led to their aggregation over time. The effect of storage conditions on the long-term stability of AgNPs was investigated and it was found that the best dispersion and stability, up to 6 months, was observed in solutions stored at 5 °C in the dark [34]. Thus, in terms of their physical and antibacterial properties, nanoparticles obtained using the biological method have similar or superior properties in comparison with the properties of nanoparticles obtained using the chemical and physical methods.

Silver nanoparticles obtained by different methods are attracting more and more attention also due to their ability to self-assemble. In the process of self-assembly, nanoparticles or other discrete objects spontaneously self-organize into ordered structures through direct and/or indirect interactions. For example, particles of uniform size can be assembled into spatially ordered structures, and the type of organization of nanoparticles and the structure of the resulting array depend on the synthesis conditions, particle diameters, as well as the nature of external influences on the structure [41]. Self-assembly processes are regulated by the balance of entropic and enthalpic effects and thus are temperature dependent [42]. Arrays of metal nanoparticles are formed through electrostatic and capillary interactions. For controlled self-assembly of nanoparticles, substrates or templates can be used to define the geometry of the system [43]. Various forms of silver nanoparticles (cubic, spherical, porous) can be synthesized from a solution of silver nitrate by self-assembly of anionic surfactants and neutral polymers in the presence of ultrasonic radiation [44]. 

**Figure 2:**
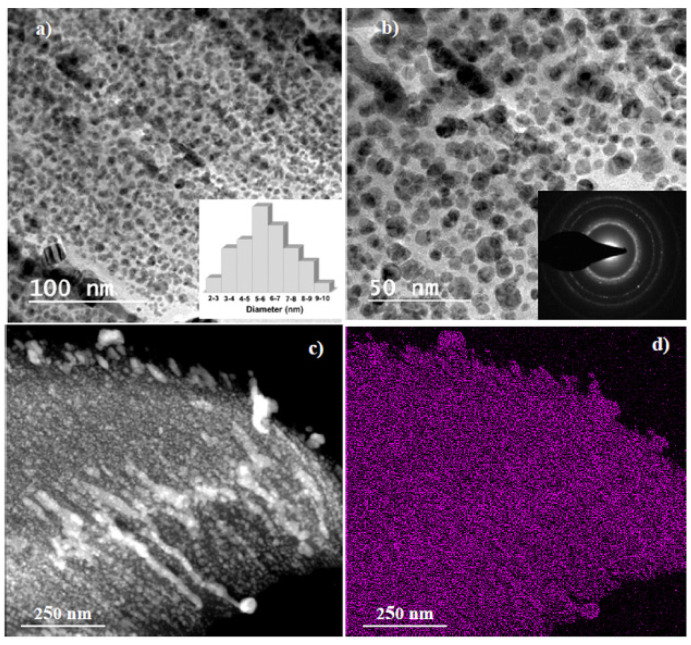
AgNPs film visualised with TEM (**a**,**b**). Inset (**a**) demonstrates size distribution and inset (**b**) is the electron diffraction pattern; Scanning energy dispersive x-ray spectroscopy mapping with (**c**) STEM image and (**d**) silver elemental mapping. Reprinted with permission from ACS Appl. Nano Mater. 2020, 3, 7, 6531–6540. Copyright © 2021 American Chemical Society [47].

Thus, self-assembly of silver nanoparticles can lead to the creation of completely new functional nanostructures or nano-organizations. To control the self-assembly process, functional polymers are used as stabilizers and organic templates. Thus, the self-assembly of silver nanoparticles on the surface of composite polymer films contributes to the formation of an interconnected three-dimensional network with increased electrical conductivity for use in LEDs [45]. For instance, for the controlled assembly of silver nanoparticles on a polyimide substrate, the method of convective self-assembly was used, and as a result, homogeneous coatings with high conductivity were obtained, which can be used as electrodes for sensors [46]. Photoinduced self-assembly of silver nanoparticles on a glass substrate allowed obtaining ultrathin AgNPs films, exhibiting high reflective and conductive properties for the use in optics and electronics [47]. A metamaterial based on silver nanoparticles with a thermosensitive coating demonstrated active self-assembly of AgNPs as a result of temperature-dependent changes in the organic coating. The spatial distribution of the tunable particles affected the optical properties of the material (Figure 2), which can be used in optical devices [48]. The use of p-aminothiophenol as a mediator provided spontaneous self-assembly of silver nanoparticles into lamellar structured nanolayers suitable for designing metamaterials [49]. A method for growing Ag/Au/Cu trimetal nanoplates using self-assembly was presented. Due to their unique structure, nanoplates can find wide application in the field of Raman scattering [50].

Most of the chemical and physicochemical methods used for the synthesis of silver nanoparticles are associated with risks and potential hazards for the environment, since they require the use of toxic reducing agents. In this regard, there is an obvious need for alternative cost-effective and at the same time safe and environmentally friendly methods for the production of nanoparticles. In recent years, a new independent direction has emerged—biogenic synthesis of nanoparticles using natural reducing and stabilizing agents [51]. Biogenic nanostructured particles are composed of both inorganic particles and biomolecules. The use of various natural sources, such as plants [52], bacteria [53], yeast [54] and other fungi [55], for the synthesis of nanoparticles has been described. Natural compounds (steroids, terpenoids, alkaloids, phenolic acid, saponins and flavonoids) that are found in various plant organs (roots, stem, bark, leaves and flowers) are used to make nanoparticles. These compounds and their metabolites play the role of reducing and stabilizing agents for the production of biogenic nanoparticles. Silver nanoparticles were prepared from stem extracts of *Ocimum sanctum* L. [56], *Coleus aromaticus* Benth. [57], *Boswellia ovalifoliolata* Bal and Henry [58] and *Piper nigrum* L. [59]. The use of various plants for the production of biogenic silver nanoparticles was described, including *Azadirachta indica A. Juss*., *Carica papaya* L., *Murraya koenigii* (L.) *Spreng*., *Ananas comosus* (L.) *Merr*., *Annona reticulate* L., *Foeniculum vulgare Mill*., *Catharanthus roseus* (L.) *G*. *Don*, *Securinega leucopyrus (Willd) Muell*, *Cardiospermum halicacabum* L., *Lawsonia inermis* L., *Camellia sinensis* (L.) *Kuntze*, *Mollugo nudicaulis Lam*., *Opuntia ficus-indica* (L.) *Mill*., *Solanum lycopersicum* L. and *Musa balbisiana Colla* [60].

**Figure 3:**
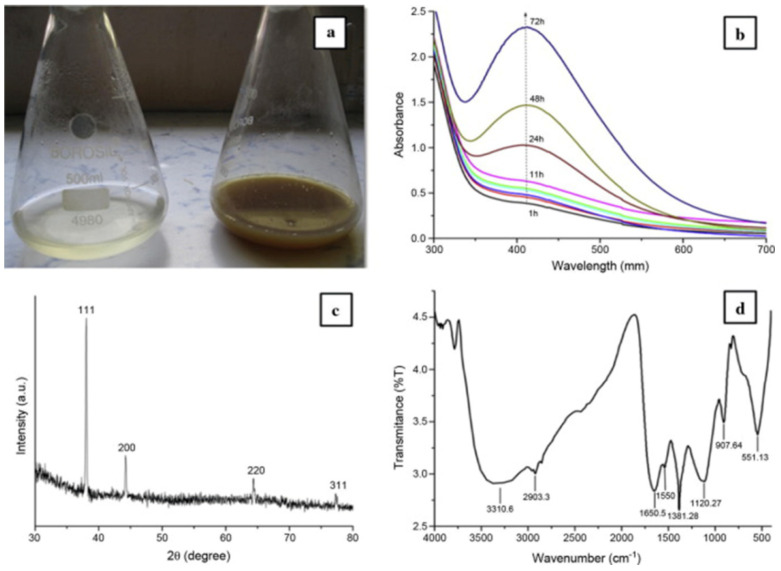
Visualization and characterization of silver nanoparticles. (**a**) The AgNPs formation; (**b**) UV–Vis spectra of AgNPs synthesized by *Rhodococcus sp.* with the absorption peak at 405 nm increasing with increased incubation time; (**c**) crystalline nature of AgNPs studied with XRD; (**d**) the FT-IR spectrum of AgNPs with the major peaks around 1550, 1650, 2903 and 3310 cm^−1^ demonstrating the presence of amides on the surfaces of AgNPs [69].

Obtaining self-assembled biogenic silver nanoparticles is more difficult due to the participation in the synthesis of various active components, such as vitamins, polysaccharides, flavones, glycosides, alkaloids, and polyphenolic compounds [61,62], however, the number of works showing the successful self-assembly of biogenic silver nanoparticles with the possibility of practical application is increasing. For instance, Zamora-Mendoza et al. [63] described the self-assembly of bimetallic Ag/Au nanoparticles (BNPs) synthesized using the plant extract of *Aloysia triphylla* Palau as a reducing agent and stabilizer. The resulting three-dimensional arrays are a consequence of the aggregation of Ag/Au nanoparticles inside the organic matrix. The three-dimensional aggregates obtained have a strong microstructure similar to crystalline materials. Aqueous seed extract of Persea americana Mill. was used as a source of bioactive molecules for self-assembly of silver nanoparticles. Avocado seed extract is rich in phenolic compounds that act as a biosurfactant, promoting the formation of particles of similar size and shape [64]. In addition to land plant extracts, biogenic synthesis of nanoparticles using algal biomass is being actively studied. It has also been shown that proteins from seaweed extract act as stabilizing and reducing agents. The alga Sargassum wightii Greville could synthesize extracellular bimetallic silver nanoparticles demonstrating excellent antibacterial properties against *Staphylococcus aureus*, *Bacillus rhizoids*, *Escherisia coli* and *Pseudomonas aeruginosa* [16]. Biogenic AgNPs synthesized using the microalgae *Chaetoceros calcitrans*, *Chaetoceros salina*, *Isochrysis galbana*, and *Tetraselmis gracilis* also showed high antimicrobial activity against human pathogens [65]. Bacteria, fungi and even viral particles are widely used for microbial synthesis of silver nanoparticles. In order to obtain hybrid nanostructures, the effect of various buffers on the self-assembly of silver nanoparticles (AgNPs) and bacteriophage M13 P9b, specific for Pseudomonas aeruginosa, was studied. Such hybrid structures can be used in tissue engineering and biomedicine for the delivery of genes and drugs [66]. For self-assembly of silver nanoparticles, a tobacco mosaic virus was used as an organic template for the controlled deposition and organization of metal nanoparticles [67]. AgNPs have specific reactivity properties when they are delivered and used in different model systems. Silver nanoparticles were synthesized on *Rhodococcus jostii* PEVJ9 bacterial cells. Self-assembly of a monolayer of silver nanoparticles on the surface of viable microbial cells enhances the biodegradation of di (2-ethylhexyl) phthalate (DEHP). Biodegradation of DEHP in the presence of AgNPs reached 100%, while without nanoparticles it was 30–66% [68]. Silver nanoparticles were synthesized intracellularly by *Actinobacteria Rhodococcus* sp. [69] and extracellularly with the help of *Bacillus subtillus* [70] (Figure 3).

The formed nanoparticles were stable in colloidal solutions and showed good bactericidal activity against pathogenic microorganisms. Biochemical changes in cyanobacteria *Spirulina platensis* and *Nostoc linckia* during the synthesis of AgNPs were studied to determine the optimal conditions for nanoparticle formation without biomass degradation [71]. 

## 3. Antibacterial and Antifungal Properties of Chemically Synthesized and Biogenic Silver Nanoparticles

The search for new antibacterial composites is urgent because of the rapid growth of drug-resistant pathogenic bacterial strains [72,73]. Pathogenic microorganisms have developed resistance to almost all types of antibiotics that are currently used [74]. In both human and veterinary medicine, bacterial resistance leads to a decrease in the effectiveness of widely used antibiotics, which may lead to further growth of infections [75]. For example, resistance to a number of drugs has already been identified in commensal bacteria such as *Escherichia coli*, in the zoonotic enteropathogens Salmonella spp., as well as in animal pathogens *Pasteurella multocida* and Actinobacillus spp. [76,77,78]. Some bacteria, for example, *Neisseria gonorrhoeae*, *Staphylococcus aureus*, and *Helicobacter pylori*, have acquired resistance to penicillin and metronidazole [79]. The presence of *H. pylori* in a patient is considered the main risk factor for the development of ulcers and stomach cancer. However, the resistance of *H. pylori* to most widely used antibiotics is growing every year all over the world [80].

**Figure 4:**
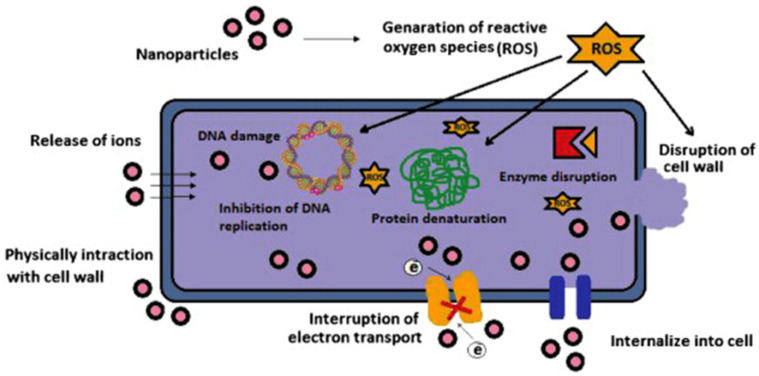
The scheme of a possible cell response to the influence of nanoparticles [83].

Since the number of available antibacterial drugs is limited, the importance of finding new antibacterial agents or cofactors enhancing the effectiveness of existing drugs increases [81]. One of the most promising directions for the development of antibacterial agents is the use of nanotechnology products [82], in particular, metal nanoparticles which have become one of the most promising options for overcoming microbial resistance and combating multidrug-resistant microorganisms (Figure 4) [72]. By now, the antibacterial activity of nanoparticles from Ag, ZnO, CuO, MgO, Si, MoO_3_, TiO_2_ and CaO has been confirmed [83,84]. Cadmium oxide nanoparticles also exhibited increased antimicrobial activity [85] while platinum nanoparticles demonstrated not only an antimicrobial activity, but were effective against cancer cells and fungi [86].

Among all metal nanoparticles, silver nanoparticles are ones of the most important due to their use as antimicrobial agents in nanomedicine [87], in groundwater treatment [88], for the manufacture of surgical masks [89], the development of wound dressings and textile fabrics for combustiology [90]. The advantage of silver nanoparticles in comparison with metallic silver or its salts is the slow and controlled release of silver ions from the nanoparticle, which provides a prolonged antibacterial effect. Microbes have a much lower ability of developing resistance to silver nanoparticles in comparison with antibiotics [91].

In turn, biogenic metal nanoparticles have shown effectiveness against drug-resistant microorganisms, both when used alone and in combination with antibiotics. Thus, biogenic silver nanoparticles synthesized using soil bacterium *Pseudomonas putida* were active against clinical isolates of *Staphylococcus aureus*, *Escherichia coli*, *Bacillus cereus*, *Pseudomonas aeruginosa*, and *Helicobacter pylori* [92]. The antibacterial effect was achieved by the nanoparticle penetration through the cell membrane, which caused the excretion of intracellular metabolites, leading to significant damage to the bacterial cell. The synthesized nanoparticles exhibited a noticeable antibacterial effect even at very low concentrations, and the growth of bacteria was inversely related to the dose used. Silver nanoparticles synthesized using an aqueous extract of the blue-green alga *Spirulina platensis*, showed high antibacterial activity against *Staphylococcus sciuri* and *Pseudomonas aeruginosa* with an inhibition zone increasing linearly with an increase in concentration of nanoparticles [93]. Biogenic colloidal silver nanoparticles synthesized with the extract of *Mentha pulegium* L. as a reducing agent demonstrated antibacterial and antifungal properties and, in addition, were cytotoxic for Hela and MCF-7 cancer cells [94].

**Figure 5:**
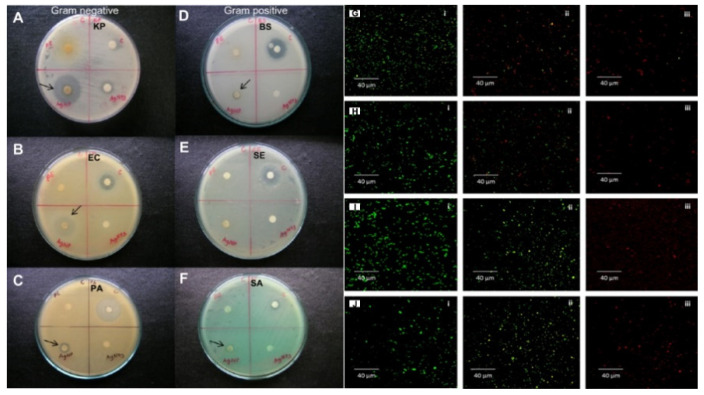
The efficacy of the aqueous extracts of Chrysanthemum indicum, AgNO3, and AgNPs against both gram-negative (**A**–**C**) and gram-positive (**D**–**F**) bacteria. Abbreviations: PE—plant extract; C—control (streptomycin); KP—*Klebsiella pneumoniae*; AgNP—silver nanoparticle; EC—*Escherichia coli*; PA—*Pseudomonas aeruginosa*; BS—*Bacillus subtilis*; SE—*Staphylococcus epidermidis*; SA—*Staphylococcus aureus*; MTCC—Microbial Type Culture Collection and Gene Bank. Reprinted with permission from Int. J. Nanomed. 2014, 9, 379–388 [82]. Confocal images of viability of bacterial cells after incubation with AgNPs and live/dead staining. Non-treated bacterial cells: (**G**i) *P. aeruginosa*, (**H**i) *E. coli*, (**I**i) *S. aureus* and (**J**i) *B. cereus*; images (ii) and (iii) demonstrate bacterial cells treated with AgNPs for 30 and 60 min accordingly [92].

Silver nanoparticles are more effective when combined with antibiotics. The combination of commercial antibiotics and silver nanoparticles synthesized by reducing silver nitrate with an aqueous leaf extract of *Epiphyllum oxypetalum* (DC.) Haworth with was more active against *Propionibacterium acne*, *Pseudomonas aeruginosa*, and *Klebsiella pneumoniae* than the nanoparticles alone [60]. Silver nanoparticles are effective at low concentrations (mg/L) as antimicrobial agents against both gram-positive and gram-negative bacteria (Figure 5) [95], selectively affect bacterial membranes [96], and are not cytotoxic for eukaryotic cells, including human erythrocytes [97]. The exact mechanism of action of colloidal silver solutions has not been clarified: the antibacterial activity of silver nanoparticles can be associated with the release of Ag+ ions or can be property of the silver nanoparticles themselves [98,99]. There are four main possible mechanisms of the antibacterial activity of colloidal silver solutions: the formation of free radicals (for example, reactive oxygen species) as a result of redox reactions, adhesion of silver NPs to the bacterial cell membrane and its destabilization, intercalation of silver nanoparticles between DNA bases with subsequent inhibition of DNA replication and transcription; and the destabilization of ribosomes, which inhibits protein synthesis.

The prevalence of fungal infections has also increased in recent years and silver nanoparticles have come to into view as potential antifungal agents. Fungal infections are most common in patients immunocompromised due to cancer chemotherapy or viral infections. They are often provoked by opportunistic strains that cause infections of the skin, nails, oral cavity and vulva. Most of these fungal diseases are caused by various *Candida* species [100]. Silver nanoparticles exhibit excellent antifungal activity against *C. albicans* by destroying cell membranes and suppressing the normal process of cell division [101]. The antifungal activity of silver nanoparticles results from formation of insoluble compounds with sulfhydryl groups in the cell wall of fungi and disruption of membrane-bound enzymes and lipids that cause cell lysis [102]. Damage to the cell wall and membrane leads to an increase in membrane permeability and the release of potassium ions (K^+^) [103]. In addition, silver nanoparticles inhibit cellular processes that are involved in yeast budding, probably, through the disruption of the membrane integrity. Transmission electron microscopy confirms the interaction between silver nanoparticles and membrane structure. During exposure to nanoparticles, *C. albicans* cells show significant surface changes, which are visualized as the formation of pits in their cell walls and pores in the cell membranes [101]. Low to moderate antifungal activity (4–8 mm ± 0.2) of biogenic silver nanoparticles synthesized using an aqueous extract of *Gymnosporia royleana* Wall leaves. ex M. A. Lawson against *C. albicans* and *Candida tropicalis* was shown [100]. Silver nanoparticles synthesized using an aqueous extract of Gymnema sylvestre R. Br. callus also exhibited significant antifungal activity against *C. albicans*, *C. nonalbicans* and *C. tropicalis* with an inhibition zone of 15.4, 14.2 and 15.7 mm, respectively. The silver nanoparticles were biocompatible, non-toxic for mammalian cells, and their antifungal activity depended on the concentration used [82]. This data is consistent with earlier studies [104,105], demonstrating that silver nanoparticles have a stronger antifungal activity than antifungal drugs. Moreover, antifungal drugs are toxic to cells at high concentrations [106]. In addition, the method of layer-by-layer self-assembly has been successfully used to create a multilayer coating on titanium substrates by electrostatic interaction between hyaluronic acid, chitosan, and silver nanoparticles. Such coatings with silver nanoparticles have antibacterial properties and are capable of destroying up to 90% of bacteria, thereby preventing infections during the installation of titanium implants [107]. Nanocomposite films with antimicrobial properties were obtained by layer-by-layer self-assembly of silver and chitosan nanoparticles on a low-density polyethylene substrate. The obtained composites, in addition to antibacterial activity, showed resistance to mechanical stress and good barrier properties, which allows their use in food packaging [108]. Silver nanoparticles biosynthesized by fungi Phenerochaete chrysosporium Burds MTCC-787 and woody oyster mushrooms Pleurotus ostreatus (Jacq.ex Fr.) P. Kumm. had a strong antimicrobial activity against Bacillus subtilis, Bacillus cereus, Staphylococichia aureus, Pseudomonas aeruginosa and a number of other pathogenic bacteria [109,110]. Silver-based hybrid biomaterials are gaining increasing attention as an alternative to traditional antimicrobial drugs. A polydopamine-coated sericin/agar composite film directed the synthesis of high-density AgNPs, resulting in an AgNPs-PDA- sericin /agar film with good antibacterial properties and high cytocompatibility with NIH/3T3 fibroblasts, which could be used as a new type of wound dressing [111]. Based on the strategy of layer-by-layer self-assembly of silver and zinc oxide nanoparticles on sericin-agarose films, an antibacterial biomaterial for tissue engineering was developed [112]. Silver nanoparticles and polyelectrolytes were used for self-assembly of AgNPs monolayer on the surface of wool fabrics. The modified fabric showed good antibacterial properties against gram-positive and gram-negative bacteria, and high durability of the antibacterial effect after repeated washings [113].

However, the use of silver nanoparticles must also be thoroughly tested to assess their safety for humans and the environment. To date, the toxicity of silver nanoparticles after prolonged intake into the human body and into the environment has not been studied in detail. However, there is evidence that chemically functionalized silver nanoparticles are more cytotoxic than biogenic ones [114]. The toxicity of nanoparticles is influenced by the method of synthesis, physicochemical properties, route of administration and duration of exposure [115,116]. In rats and mice, the following safe doses of silver nanoparticles were found oral dose 0.5 mg/kg (3–40 nm), intravenous dose 2 mg/kg (10–14 nm), inhalation dose 0.1 g/kg (15–19 nm) and a local dose of 10 μg/kg (30–34 nm), which is a good indicator that these concentration ranges, when recalculated for humans, will be quite safe [117]. In addition, there is no significant toxicity of silver nanoparticles evidenced by the level of ALT, AST, Gamma-glutamyltransferase (biomarkers of liver function) and creatinine (a biomarker of kidney function) in the blood serum of rats. In studies by Gengan et al. it was found that silver nanoparticles are not toxic to peripheral lymphocytes of a healthy individual [118,119]. However, careful further toxicity studies of silver nanoparticles obtained by different methods are required.

## 4. Comparative Efficacy and Toxicity of Silver Nanoparticles Obtained by Different Methods of Synthesis

Several comparative analyses of therapeutic potentials of biogenic and chemically synthesized silver nanoparticles were carried out. In the study of antileishmanial activity, biogenic silver nanoparticles turned out to be more effective and significantly reduced the pathogenicity of parasites, in contrast to chemically synthesized AgNPs [120].

Silver nanoparticles synthesized using a leaf extract of medicinal plant *Nathophodytes foetida* exhibited a strong cytotoxic effect on human leukemic cells (K562), along with chemically synthesized nanoparticles [121]. The nanoparticles biosynthesized by *Fusarium oxysporum* and *Azadirachta indica* were polydisperse, 10–40 nm in size, and the chemically synthesized ones were monodisperse, 5 nm in size [122]. Antimicrobial analysis showed that the biogenic nanoparticles had better antibacterial properties against *E. coli* and *S. aureus,* probably because of higher protein capping of biogenic nanoparticles facilitating their entry into bacterial cells [122]. Other authors explained higher antibacterial activity of biogenic AgNPs by synergistic antibacterial effect of the nanoparticles and plant extract metabolites, adsorbed on them [123]. In their work, biogenic silver nanoparticles synthesized using *Datura stramonium* L. leaf extract had a spherical shape and a narrow size range, and demonstrated high antibacterial and DNA-cleaving activity, as well as antioxidant properties. Chemically synthesized AgNPs were smaller but did not possess antioxidant properties and had weaker antibacterial and DNA-cleaving activity [123]. When a plant culture used for biogenic nanoparticle synthesis had little or no antimicrobial activity, size differences could be responsible for better performance of green-AgNPs compared to the chemical AgNP [124]. Thus, silver nanoparticles synthesized by *Elettaria cardamomum* leaf extract demonstrated lower minimal inhibitory concentration when tested on a number of fungal phytopathogens, in comparison with chemical AgNPs. The fungicidal effect of both AgNPs types further increased in combination with fungicides (carbendazim, mancozeb, and thiram) [124].

Moreover, biosynthesized silver nanoparticles have a higher biocompatibility compared to chemically synthesized AgNPs [125]. The leaf extract of *Millettia pinnata* (L.) Panigrahi was used as a reducing agent for the biosynthesis of silver nanoparticles. Capping of biogenic AgNPs by polyphenolic compounds present in the leaf extract provided high antioxidant activity of the nanoparticles and significantly reduced their toxicity compared to chemically synthesized AgNPs [126]. The influence of biogenic and chemically synthesized silver nanoparticles on the structure of benthic bacterial communities was studied. Thermoleophilia bacteria were resistant to both forms of AgNPs, while Koribacteraceae bacteria were sensitive [127]. A comparative toxicity study of chemically and biologically synthesized silver nanoparticles towards the plant *Solanum lycopersicum* L. showed that biogenic AgNPs were less toxic than the chemically synthesized ones [128]. Begum et al. studied the effect of biogenic and chemically synthesized silver nanoparticles on the biomass accumulation of callus cultures of *Fagonia indica* Burm. Biogenic AgNPs, were more biocompatible, producing larger biomass of *Fagonia indica* Burm. [129]. In studies with *Bacillus subtilis* cultures, biogenic AgNPs synthesized using the *Allium cepa* L. extract were significantly less toxic to normal human gut microbiota than chemically synthesized ones [130]. The effect of various concentrations of biologically and chemically synthesized silver nanoparticles was studied in freshwater fish *Oreochromis niloticus* L. A higher level of expression of the heat shock protein (HSP70) was observed in all tissues of fish exposed to chemically synthesized silver nanoparticles, compared with biologically synthesized ones [131].

## 5. Conclusions

Biogenic silver nanoparticles are environmentally safer compared to particles obtained by chemical or physical methods due to the absence of toxic compounds in the technological process, gentle synthesis conditions and the possibility of utilizing the biomass used in their production. Biogenic silver nanoparticles are capable of self-assembly, including that on various surfaces, and there are mechanisms for controlling their morphology and size. Approaches and methods for obtaining biogenic silver nanoparticles using various parts of plants, algae, fungi and microorganisms have been described. It has been shown that biogenic silver nanoparticles have pronounced antimicrobial and antifungal activities, which are due to the special mechanisms of the effect of active forms of silver on pathogenic organisms. These special properties open up the possibilities for the application of technologies using non-toxic silver nanoparticles of biogenic origin as an alternative or addition to antibiotics and antifungal drugs under conditions of constantly growing resistance of pathogens to the applied conservative therapy.
